# Quantitative volumetric imaging of normal, neoplastic and hyperplastic mouse prostate using ultrasound

**DOI:** 10.1186/s12894-015-0091-9

**Published:** 2015-09-21

**Authors:** Shalini Singh, Chunliu Pan, Ronald Wood, Chiuan-Ren Yeh, Shuyuan Yeh, Kai Sha, John J. Krolewski, Kent L. Nastiuk

**Affiliations:** Departments of Pathology and Laboratory Medicine, University of Rochester School of Medicine and Dentistry, Rochester, NY USA; Departments of Neurobiology and Anatomy and Obstetrics and Gynecology, University of Rochester School of Medicine and Dentistry, Rochester, NY USA; Department of Urology, University of Rochester School of Medicine and Dentistry, Rochester, NY USA; Current address: Department of Cancer Genetics, Roswell Park Cancer Institute, Buffalo, 14263 NY USA

**Keywords:** Prostate cancer, BPH, Mouse model, IVIS, 3D volume

## Abstract

**Background:**

Genetically engineered mouse models are essential to the investigation of the molecular mechanisms underlying human prostate pathology and the effects of therapy on the diseased prostate. Serial *in vivo* volumetric imaging expands the scope and accuracy of experimental investigations of models of normal prostate physiology, benign prostatic hyperplasia and prostate cancer, which are otherwise limited by the anatomy of the mouse prostate. Moreover, accurate imaging of hyperplastic and tumorigenic prostates is now recognized as essential to rigorous pre-clinical trials of new therapies. Bioluminescent imaging has been widely used to determine prostate tumor size, but is semi-quantitative at best. Magnetic resonance imaging can determine prostate volume very accurately, but is expensive and has low throughput. We therefore sought to develop and implement a high throughput, low cost, and accurate serial imaging protocol for the mouse prostate.

**Methods:**

We developed a high frequency ultrasound imaging technique employing 3D reconstruction that allows rapid and precise assessment of mouse prostate volume. Wild-type mouse prostates were examined (*n* = 4) for reproducible baseline imaging, and treatment effects on volume were compared, and blinded data analyzed for intra- and inter-operator assessments of reproducibility by correlation and for Bland-Altman analysis. Examples of benign prostatic hyperplasia mouse model prostate (*n* = 2) and mouse prostate implantation of orthotopic human prostate cancer tumor and its growth (*n* = 6) are also demonstrated.

**Results:**

Serial measurement volume of the mouse prostate revealed that high frequency ultrasound was very precise. Following endocrine manipulation, regression and regrowth of the prostate could be monitored with very low intra- and interobserver variability. This technique was also valuable to monitor the development of prostate growth in a model of benign prostatic hyperplasia. Additionally, we demonstrate accurate ultrasound image-guided implantation of orthotopic tumor xenografts and monitoring of subsequent tumor growth from ~10 to ~750 mm^3^ volume.

**Discussion:**

High frequency ultrasound imaging allows precise determination of normal, neoplastic and hyperplastic mouse prostate. Low cost and small image size allows incorporation of this imaging modality inside clean animal facilities, and thereby imaging of immunocompromised models. 3D reconstruction for volume determination is easily mastered, and both small and large relative changes in volume are accurately visualized. Ultrasound imaging does not rely on penetration of exogenous imaging agents, and so may therefore better measure poorly vascularized or necrotic diseased tissue, relative to bioluminescent imaging (IVIS).

**Conclusions:**

Our method is precise and reproducible with very low inter- and intra-observer variability. Because it is non-invasive, mouse models of prostatic disease states can be imaged serially, reducing inter-animal variability, and enhancing the power to detect small volume changes following therapeutic intervention.

## Background

Prostate cancer (PrCa) is the most prevalent non-cutaneous cancer and second leading cause of cancer mortality in men [1]. Despite effective therapy for localized disease, treatment of recurrent and metastatic PrCa is problematic and therefore the focus of intense investigation. Mouse models have proven valuable for studying human disease, including prostate cancer, because of their short breeding cycles and manageable costs, but particularly because of the ease of genetic manipulation compared to larger animals. Mice are also useful as immunocompromised hosts for xenografts of human prostate cancers [2, 3]. Genetically engineered mouse models have been particularly informative in revealing the molecular and cellular mechanisms underlying prostate tumor biology, and to evaluate new therapeutic inventions [4]. Although fewer models are available, benign prostatic hyperplasia can also be genetically engineered in mice, which may be useful in developing therapies for this highly prevalent disease [5].

US Food and Drug Administration approval rates for drugs has been declining since 1990 and oncology drug development has been particularly inefficient [6]. Only about one in twenty drugs entering phase I human clinical trials for cancer are eventually approved and among those that progress to phase III trials, only about 30 % are eventually approved [6]. A major cause of this failure is lack of efficacy in human trials, rather than safety issues. Specifically, the apparent efficacy in murine models is frequently not replicable in human trials [7, 8]. To enhance reproducibility, and ultimately the translation of pre-clinical trial success into human clinical trials, it has been proposed that all pre-clinical trials include randomization of tumor bearing animals to treatment groups; blinding to those treating, and subsequently evaluating, endpoints; a pre-determined statistical analysis protocol; and treatment designs that are adequately powered to test the null hypotheses [9, 10]. Accurate volumetric imaging serves an important role in executing such rigorous preclinical trials by ensuring that all animals undergoing randomization to treatment groups have tumors of similar size. This is particularly important in models where tumor formation does not occur in 100 % of mice or where there is significant variation in the rate of tumor formation [11]. In addition, live animal imaging of tumor volume provides an accurate assessment of tumor response kinetics, controlled for animal-to-animal variation, since each animal serves as its own control [12]. The alternatives – serially sacrificing groups of animals at intermediate time points, or simply measuring tumor size at the end of the trial – are less informative or require many more animals, compromising the statistical power of the preclinical study design [13].

While rodent and human prostates are functionally equivalent, the mouse prostate is small (~25 mm^3^), multi-lobular (ventral, dorsal, lateral and anterior), and interdigitated with surrounding genitourinary organs. Many reports have monitored prostate tumor growth and regression using optical imaging (fluorescence or luciferase reporters). However, tumors must be engineered to express a reporter gene and quantitation is very problematic [14, 15], particularly for longitudinal imaging [16, 17]. Optical imaging is both higher throughput and lower in cost than other modalities, but has relatively poor anatomic resolution [18]. We have previously described a quantitative and reproducible anatomical imaging approach, utilizing high-field magnetic resonance imaging with chemical shift suppression (MRI-CHESS) [19, 20] to measure murine prostate volumes changes resulting from a variety of manipulations in mouse models. Since MRI instrumentation is costly and not widely available, we sought to develop a 3D-ultrasound imaging protocol to precisely measure very small changes in the volume of the mouse prostate, in living animals.

High-frequency ultrasound imaging of the mouse prostate has many advantages: it is low cost and high throughput, enables 3D reconstruction for precise volume determination, and allows real-time imaging to facilitate surgical manipulations [21]. Here, we describe the use of high-resolution *in vivo* ultrasound imaging for quantitative analysis of prostate volume. Monitoring changes in the prostate of normal mice, we demonstrate that this ultrasound technique can precisely detect sub-cubic millimeter changes in volume following hormonal manipulations that result in regression and regrowth of the normal prostate. We then apply this technique to two distinct prostate disease models: growth of the prostate in a prolactin-driven benign prostatic hyperplasia model [22], and growth of human prostate cancer orthotopic xenografts implanted in the anterior lobe of the mouse prostate [23].

## Methods

### Animals

All animal studies were approved by the University of Rochester institutional animal use committee (UCAR #2012-030) and conducted according to the guidelines of the local committee on animal resources, as well as all relevant national guidelines. Six to nine month old male C57/BL6 mice, and 12–14 week old male nude mice, used as orthotopic xenograft hosts, were purchased from Charles River Labs. Probasin-driven prolactin (Pb-PRL) transgenic mice were provided by John Kindbloom [22] and backcrossed into an FVB background [24]. All mice were housed individually and provided food and water *ad libitum*. Endocrine perturbation was as described previously [19]. Briefly, animals were anesthetized via intra-peritoneal Ketamine (87 mg/kg) and Xylazine (13 mg/kg) injection and then surgically castrated via scrotal incision. Testes were removed, and blood vessels and vas deferens ligated. The incision was closed with wound clips. Three days following castration clips were removed to allow ultrasound imaging. Fourteen days following castration, 5 mg/kg 5alpha-androstan-17beta-ol-3-one (dihydrotestosterone, DHT, Sigma) in corn oil was injected sub-cutaneously daily.

### High-resolution ultrasound image acquisition and analysis

Each mouse was imaged with a high-resolution ultrasound system (Vevo770 high resolution imaging system, VisualSonics, Toronto, Ontario, Canada) using the highest resolution scan head (either 710 or 704b) that was able to image the entire prostate or tumor. The 704b scan head probe is driven by a linear motor with a center frequency of 40 MHz and provides a 40 μm axial and 80 μm lateral resolution at a focal depth of 6 mm, affording a 14.5 μm field of view. The corresponding values for the 710 scan head are 25 MHz, 70 μm × 140 μm, and 20.8 μm, respectively. Mice were anesthetized in a chamber using 3 % isoflurane and then fixed in the transverse position on a heated imaging platform (Vevo Integrated Rail System III, VisualSonics) with a nose cone for maintenance of anesthesia using 2 % isoflurane. Animals were monitored for heart rate and respiratory cycle using surface electrodes. The abdomens of the mice were depilated using a commercial calcium thioglycolate product (“Nair”), and ultrasound gel (Aquasonic 100, Parker Laboratories, Fairfield, NJ) was applied to the abdomen. The location of the anterior, dorsal and ventral prostate lobes was identified by mechanically adjusting the position of the ultrasound transducer, with the bladder and urethra as landmarks. Images of 585 sections of each ultrasound series were acquired at a resolution of 1600 × 1200 pixels using the VisualSonics software. Following acquisition, images were imported into Amira software (Visualization Sciences Group, Burlington MA), for 3D mouse prostate volume reconstruction. The images were set to 8-bit gray scale and the contrast enhanced using Amira. Anatomic boundaries of the prostate lobes were manually outlined in parallel slices. Based on these areas, the volume was subsequently computed with Amira. Slice alignment, segmentation and generation of surface meshes were also performed with Amira. Volume, in cubic millimeters, or mean volumes, accompanied by standard error of the mean (SEM) if multiple equivalent imaging sessions were analyzed, are presented. In the reproducibility study, the coefficient(s) of variation (CV) was calculated as the appropriate standard deviation divided by the mean, and expressed as a percentage. For all other studies, a single determination is presented.

### Cell culture

For orthotopic prostate tumor implantation in nude mice, CWR22Rv1 cells [25] were acquired from American Type Culture Collection (Manassas, VA) and grown in Roswell Park Memorial Institute 1640 (RPMI) medium containing glutamine and antibiotics with 10 % fetal calf serum. Prior to injection, cells were washed with phosphate buffered saline, harvested with trypsin/Ethylenediaminetetraacetic acid solution, and pelleted. Cells were re-suspended in RPMI media and an equal volume of Matrigel (Corning, Bedford, MA) was added.

### Image-guided orthotopic prostate tumor establishment

Nude mice were imaged using high-frequency ultrasound, as above, prior to tumor xenografting to establish baseline images. For implantation, image acquisition was performed with the 704b probe using enhanced abdominal visualization in 3D-mode. Physiological status (electrocardiogram, respiration, blood pressure, and body temperature) of the mice was monitored during each image-guided injection. While monitoring these images, a 30-gauge needle on a syringe was positioned with a micro-manipulator into the junction of the two lobes of the anterior prostate. A volume of 10 μL containing 1x10^6^ tumor cells in 50 % Matrigel was injected over 20 s. The syringe was then withdrawn, ultrasound gel removed, and mice allowed to recover. Mice were imaged weekly for eight weeks to monitor development of the orthotopic prostate tumor xenografts.

### Statistical analysis

Prostate volume was normalized to pre-castration volume for each animal and the mean of group of each time (+/− SEM), relative to pre-castration volume is presented in each data set. Comparison between groups was analyzed by one-way ANOVA with Dunnetts’s post-hoc test. Subsets of the data were quantitated independently by two blinded observers to create a Bland-Altman plot [26, 27]. The means of the prostate volumes, generated by two independent observers, were plotted on the horizontal axis, and the differences between the two observers were plotted on the vertical axis. Differences were considered statistically significant for a value of *P* < 0.05.

## Results

### Ultrasound imaging of WT mouse prostate

**Fig. 1:**
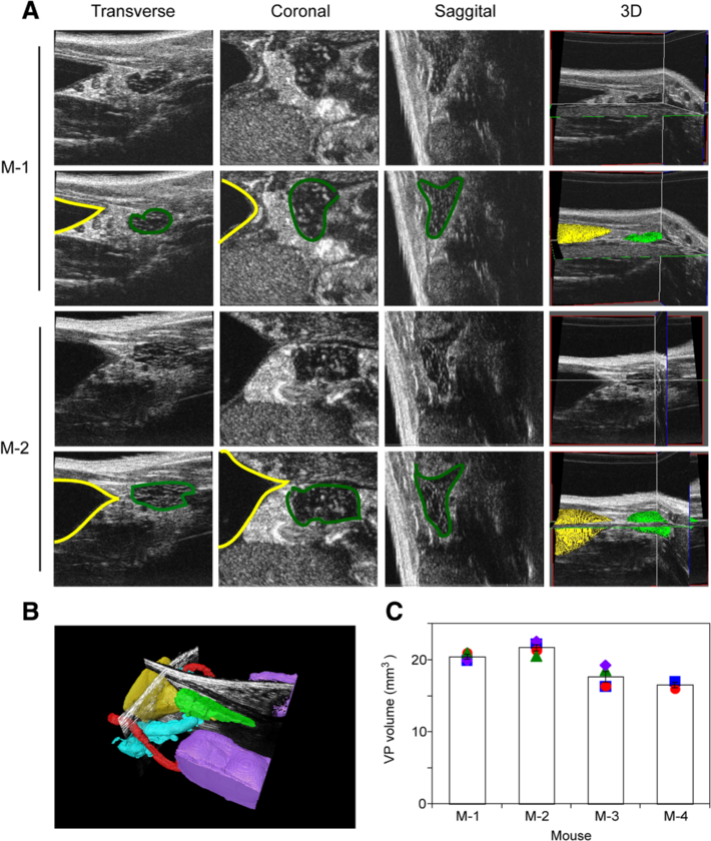
Ultrasound volume quantitation of mouse ventral prostate is highly reproducible. **a** Upper sets of panels (for mice M-1 and M-2) depict the ultrasound images of the lower genitourinary system resolved into three orthogonal planes (transverse, coronal and sagittal, as indicated) and the corresponding integrated 3D images. The lower set of panels (for mice M-1 and M-2) illustrates the process of segmentation of the relevant anatomic structures (*bladder in yellow*, *ventral prostate in green*) using the Amira software. **b** Amira generated segmented ultrasound image of the lower genitourinary system from mouse M-1, including bladder (*yellow*), seminal vesicle (*blue*), testes (*purple*), ventral prostate (*green*) and vas deferens (*red*). **c** The ventral prostate volume for each of four mice was repeatedly determined. Each symbol represents an independent determination for a given mouse. Columns denote the mean and error bars the SEM. M-1, M-2 and M-3 were imaged four times, while M-4 was imaged twice.

Prostates of four normal mature mice were imaged using high frequency ultrasound to assess organ volume. Images of three orthogonal (transverse, coronal and sagittal) planes of the ventral prostate (VP) were acquired and integrated 3D images produced using VisualSonics software. The rodent prostate gland is composed of multiple lobes: ventral, dorsal, lateral and anterior, which are interdigitated into surrounding tissues [28], while in humans the gland is non-lobular (unitary). The mouse VP has a larger epithelial component and therefore a larger volume of luminal prostatic secretions relative to the dorsal-lateral prostate (DLP) lobes, which share ducts and have similar proportions of epithelial and stromal components, and therefore are often considered together. As the VP is most echogenically distinct, segmentation of this lobe is most accurate, and henceforth reported when describing normal prostate volume changes. Image sets for two of these mice, M1 and M2, are illustrated in Fig. 1a. 3D images were imported into Amira visualization software, manually outlining the bladder (yellow) and VP (green) in all three planes (Fig. 1a, lower set of panels for each mouse). Using the segmentation illustrated in Fig. 1a, Amira was employed to produce 3D reconstructions of these organs, as well as the seminal vesicles and vas deferens (Fig. 1b). To assess the reproducibility of this method for determining the volume of the VP, the same four mice were each imaged four times. Based on the corresponding segmentation and 3D reconstruction, the volumes of the ventral lobes of these mouse prostates were computed using Amira (Fig. 1c). The average VP volume for these mice was 19.03 +/− 3.01 mm^3^; (mean +/− SEM, *n* = 4). The intra-mouse variability was quite low (CV% = 5.0 %), suggesting that for a mature mouse a single imaging session is sufficient to determine the baseline prostate volume for further studies that measure organ volume regulation.

### Regression of WT prostate volume after castration

**Fig. 2:**
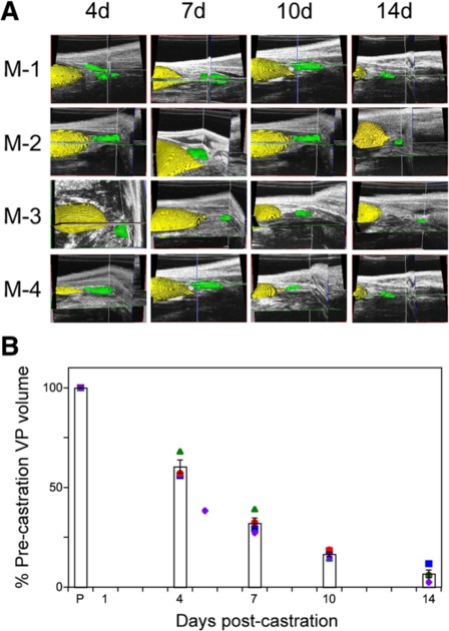
Castration induced prostate regression monitored by high-resolution 3D ultrasound imaging. Imaging of VP volume in four mice, post-castration. **a** Amira generated 3D volume reconstructions from ultrasound images, for four mice (M-1 through M-4). Segmentation of the bladder (*yellow*) and the ventral prostate (*green*) is illustrated. **b** Plot of ventral prostate volume regression. VP volume was normalized to pre-castration (P) volume for each animal and the mean (columns, ± SEM) of the group at the indicated day, relative to pre-castration volume. Symbols correspond to M-1 (*square*); M-2 (*circle*); M-3 (*diamond*) and M-4 (*triangle*).

Advanced, recurrent, and metastatic prostate cancer is typically controlled using androgen deprivation therapy (ADT) [29]. ADT induces apoptosis of the epithelial cells, resulting in regression of normal and diseased prostate glands as well as disseminated tumor in both patients and the corresponding mouse models [3, 28, 30]. During castration, the VP undergoes greater regression than other lobes due to the relatively higher proportion of epithelial cells [19, 31, 32]. To determine the ability of high frequency 3D ultrasound to monitor prostate regression, four WT mice were surgically castrated and VP volume assessed every 3 or 4 days thereafter by ultrasound imaging. We observed significant reduction of VP volume in all mice from day 4 through day 14 (Fig. 2a). Amira derived volumes indicate that by day 4, the ventral prostate lobes regressed 39 ± 3 %. This progressed to 68 ± 3 % on day 7 and was further reduced to 84 ± 2 and 92 ± 2 % on day 10 and 14, respectively, relative to the corresponding mouse’s intact VP volume (P < 0.001, Fig. 2b). The normalized CVs (relative to the intact VP volume for each mouse, “intra-mouse CV”) were 6.8 %, 5.3 %, 2.2 %, 3.8 % for days 4, 7, 10, and 14, respectively, much smaller than the overall VP volume variance of 12.6 % (“inter-mouse CV”) when not normalized to pre-castration volume, as would be the case if one were to examine VP volumes for groups of mice sacrificed serially.

### Regrowth of ventral prostate volume in normal mice

**Fig. 3:**
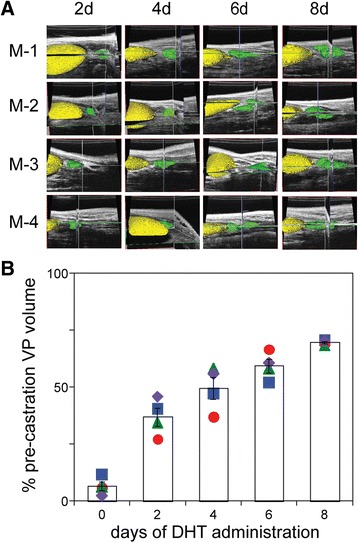
Ventral prostate lobe re-growth in previously castrated mice, following administration of exogenous DHT. **a** Amira generated 3D volume reconstructions from ultrasound images, of four mice (M-1 through M-4), acquired 2, 4, 6 and 8 days following the administration of DHT to the cohort of day 14 castrated mice shown in Fig. 2 (corresponding to the mice in the right-most column of B). Segmentation of the bladder (*yellow*) and the ventral prostate (*green*) is illustrated. **b** Plot of ventral prostate volume, following DHT administration, over time (days). Volumes determined at each time point were normalized to the pre-castration (intact) volume (in Fig. 2). Columns denote the mean and error bars correspond to the SEM. Symbols correspond to M-1 (*circle*); M-2 (*diamond*); M-3 (*square*) and M-4 (*triangle*).

The prostate of castrated mice returns to its pre-castrated size following 2 to 3 weeks of androgen supplementation [19, 31]. Two weeks following castration, the four mice depicted in Fig. 2 were supplemented daily with DHT and changes in prostate volume were monitored using high frequency 3D ultrasound imaging. Following manual segmentation and 3D reconstruction using Amira software to delineate the bladder (yellow pseudocolored structure in Fig. 3a) and ventral prostate (green structure in Fig. 3a), we calculated the volume of the VP for these mice. There was a steady increase in the ventral prostate volume, from 8 % of the pre-castrated volume at the beginning of DHT treatment, to 36.4 ± 5, 49.57 ± 4, 59 ± 5, and 70 ± 0.5 % on days 2, 4, 6, and 8, respectively. This represents regeneration of two-thirds of the VP volume in eight days (Fig. 3b). The variability of this regeneration appears to be higher at intermediate time points (21.7 % CV at day 2, 16.7 % CV at day 4, 13.6 % CV at day 6) compared to the final measurement (2.4 % CV at day 8), suggesting that this represents underlying biological variability in the rate of regrowth, rather than reflecting inherent variability in our imaging technique.

### Intra- and inter-observer variability

**Fig. 4:**
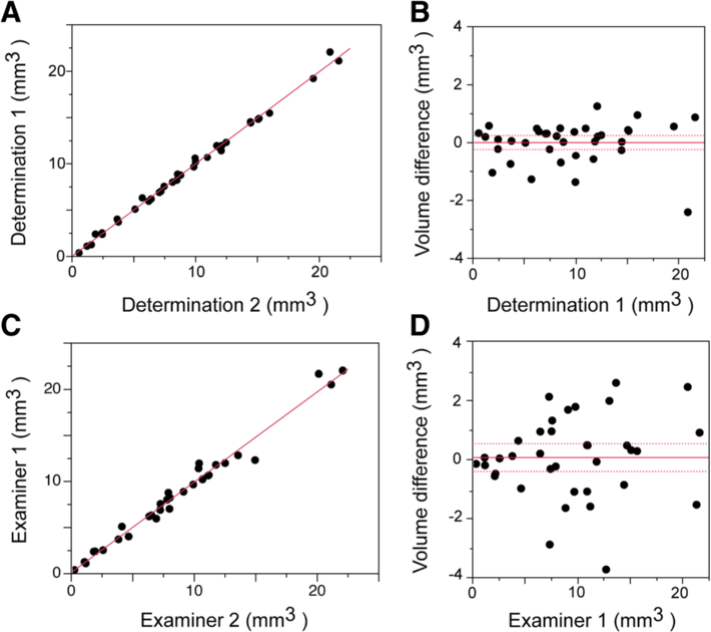
Intra- and inter-observer variability of volume measurement by ultrasound imaging. Intra-observer variation (**a**, **b**). **a** Linear correlation plot of two independent sets of volume measurements, performed (blinded) by a single observer (data from Figs. 2 and 3 image sets). The correlation coefficient, r^2^ = 0.995. **b** Bland-Altman plot of the data from (**a**). Dashed lines correspond to the 95 % confidence interval. The mean deviation of individual measurements was 0.8 % (*n* = 36). Inter-observer variation (**c**, **d**). **c** Linear correlation plot of two independent sets of volume determinations, performed (blinded) by two separate examiners (data from Figs. 2 and 3 image sets). The correlation coefficient, r^2^ = 0.945. **d** Bland-Altman plot of the data from (**c**). Dashed lines correspond to the 95 % confidence interval. The mean deviation of individual measurements was 3.0 % (*n* = 32).

To examine the precision of high frequency ultrasound measurement of the mouse VP volume, the images generated in Figs. 2 and 3 were blinded and reanalyzed by the primary reviewer and a secondary reviewer [20, 33–35]. Following segmentation and 3D reconstruction, the volumes were recorded, sent to an honest broker, decoded, and the intraclass correlation coefficients and variability for intra- and inter-observer assessments were calculated [33]. Intra-observer correlation was quite robust, with r^2^ = 0.995 (Fig. 4a). The mean intra-observer deviation of individual measurements, as measured by Bland-Altman analysis was 0.8 % (Fig. 4b), suggesting strong confidence in the reproducibility of the high frequency ultrasound 3D reconstruction of the VP volume. In addition, this data was blinded and quantitated by two independent observers and inter-observer agreement analyzed by Bland Altman analysis (Fig. 4c and d). Volume correlation for the VP was r^2^ = 0.945 (Fig. 4c), and the mean deviation 3.0 % (Fig. 4d). The interclass correlation coefficient for measurements of volume between the first and second examiner also revealed that the intra- and inter-observer variability for regression of the ventral prostate was 2.5 and 6 %, respectively, indicating that differences between the groups of mice were not due to observer bias.

### Ultrasound imaging of hyperplastic mouse prostate

**Fig. 5:**
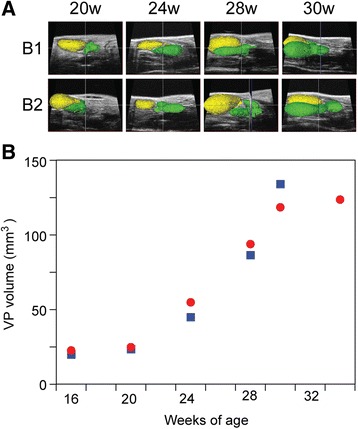
Quantitative monitoring of a benign prostatic hyperplasia in probasin-PRL transgenic mice. **a** Amira generated 3D reconstructions from ultrasound images, for two probasin-PRL mice (B-1 and B-2), acquired at 30, 34, 38 and 41 weeks of age. Segmentation of the bladder (*yellow*) and the ventral prostate (*green*) is illustrated. **b** Plot of ventral prostate volume over time (age, in weeks). Symbols correspond to the same animal imaged serially.

The volume of the ventral prostate was monitored during development in a mouse model of benign prostatic hyperplasia using high frequency ultrasound imaging. Pb-PRL transgenic males developed a significant enlargement of the prostate gland, characterized primarily by hyperplasia of the stromal compartment, distended ductal structures, and focal areas of glandular dysplasia [22]. Two Pb-PRL mice were monitored from 16 to 30 weeks of age, and one of these an additional four weeks. Ventral prostate and bladder were manually segmented and boundaries in parallel slices and Amira three-dimensional reconstructions are shown in Fig. 5a. Ultrasound images of the hyperplastic prostates were acquired using the 710 probe for the wider field of view. Computed VP volumes of the Pb-PRL transgenic mice from 16 to 30 weeks (and 34 weeks for mouse B1) are plotted in Fig. 5b. Fortuitously, an animal care issue unrelated to prostate volume required that a single Pb-PRL mouse (not one of the two monitored in Fig. 5) be sacrificed at 28 weeks of age, and so the wet weight of micro-dissected ventral prostate was then determined to be 80 mg, while 3D reconstruction of high frequency ultrasound of this mouse abdomen, performed on the same day, resulted in a prostate volume of 82 mm^3^ (data not shown). Prolactin-driven prostate volume increases rapidly from 20 weeks of age, and slows by 30 weeks of age, while continuing to grow slowly thereafter (data not shown). Unfortunately, attempts to microdissect the BPH prostates at later ages proved unsuccessful due to the interdigitated nature of the diseased prostate into the surrounding tissues.

### Development of orthotopic xenografts

**Fig. 6:**
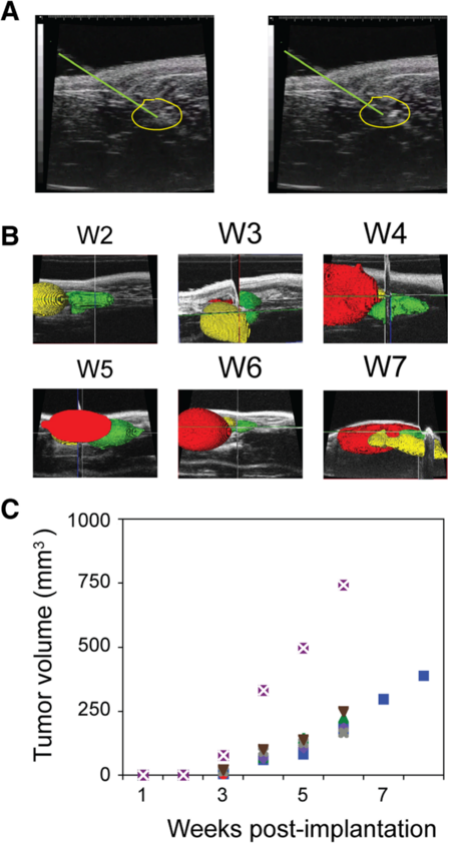
Monitoring growth of orthotopically implanted human prostate cancer xenograft. **a** Ultrasound image demonstrating 30-gauge needle injection (needle track is above and to the right green line) of 10^6^ CWR22Rv1 castration resistant prostate cancer cells into the murine anterior prostate lobe (*yellow outline*), before (*left*) and after (*right*) injection of the cells. **b** Amira generated 3D volume reconstructions from ultrasound images over time (weeks). Segmentation of the xenograft tumor in the anterior prostate (*red*), the bladder (*yellow*) and the ventral prostate (*green*) is illustrated. Images in upper three panels were acquired with a 704 probe (80 mm FOV); lower images were acquired with a 710 probed (120 mm FOV). **c** Plot of orthotopic tumor volume increase over time (age, in weeks). Symbols correspond to the same animal imaged serially.

We used high frequency ultrasound imaging to guide the establishment and monitor the growth of an orthotopic human prostate cancer mouse xenograft model. The genitourinary anatomy of nude mice was imaged by high resolution, high frequency ultrasound (using the VisualSonics 704 probe) and the junction of the anterior prostate lobes was identified. A syringe needle was then aligned with the junction and advanced into a lobe of the anterior prostate (circled in Fig. 6a) using the needle guide overlay feature of the VisualSonics software that allows for the simultaneous visualization of the needle alignment and injection target on the monitor. CWR22Rv1 cells in matrigel were injected directly into a lobe of the anterior prostate and were visualized post-injection as the hyperechoic signature at the end of the hypoechoic needle displacement track in Fig. 6a (right side). Non-invasive high frequency ultrasound imaging was used to identify the site of origin of the tumor and then to serially monitor growth of the CWR22Rv1xenografts. We observed tumor growth starting at week 3 in one lobe of the anterior prostate (volume approximately 10 mm^3^; Fig. 6b, “W3” and by the fourth week tumor volume increased rapidly, reaching the field of view limit of the 704 probe to simultaneously visualize the entire tumor volume (approximately 60 mm^3^; Fig. 6b, “W4”). Tumor growth was further monitored using the 25 MHz frequency 710b probe from week 5 to week 7 post-implantation (Fig. 6b, bottom) or until tumor volume reached 750 mm^3^, when the xenograft hosts were sacrificed. For the six xenografts in this proof of principle, we observed most xenografts grew at a rate of ~50 mm^3^ per week, while one xenograft had a much more aggressive growth phenotype (doubling every week, Fig. 6c).

## Discussion

Androgens regulate prostate growth and neoplastic phenotype. They promote mitosis and differentiation of rodents prostate ductal epithelium, and further inhibit apoptosis of differentiated cells [36]. Androgen withdrawal, through surgical castration or pharmacological blockade, induces apoptosis of the ductal secretory epithelium, but not the basal epithelium or stromal cells [37]. The small size of the mouse prostate (25 mm^3^) versus the human prostate (25 cm^3^) and the difficulty in reproducibly excising the prostate from surrounding tissues makes it challenging to accurately determine prostate volume by weighing or histological analysis at necropsy [3, 19, 20, 38–40]. Moreover, as noted in the Introduction, restricting analysis to animals at necropsy would require large numbers of animals for longitudinal studies of tumor or BPH response to therapy and is inherently less accurate than repeated volumetric imaging of individual animals over time. Biochemical cancer biomarkers, such as serum PSA for human prostate xenograft volume [11, 41], and serum PSP94 for mouse tumor volume [42], correlate well with tumor volume but are androgen driven, and therefore problematic for monitoring therapies directed at androgen signaling. Thus, there is a need for a relatively rapid and inexpensive, yet quantitative, methodology for imaging the murine prostate.

In 2006, Albanese and colleagues [43] pioneered the use of high field strength MRI to measure prostate volume in mouse tumor models. Subsequently, we developed [19] and utilized [20] a quantitative and reproducible magnetic resonance-based anatomical imaging approach (MRI-CHESS) to quantitate murine prostate volume changes in mouse models. CHESS (chemical shift suppression) allows suppression of MR signal arising artifactually from surrounding peri-prostatic fat, revealing boundaries of the prostatic lobes more accurately, and differentiating the ventral from the dorsal-lateral lobes. However, since MRI requires costly instrumentation, with high operating costs, and the additional expense of a dedicated operator [44], it is not available to the vast majority of prostate cancer investigators. Moreover, achieving the very high spatial resolution required to accurately determine prostate volumes with MRI necessitates long scan times, and therefore this methodology is decidedly low throughput. Finally, the significant infrastructure required to support an MRI facility often precludes location within the clean zone of an animal facility, which hampers the ability to image immunocompromised animals (such as xenograft-bearing athymic nude mice) which require a pathogen-free environment.

Given these obstacles to the implementation of MRI, and in the absence of other well-characterized alternative quantitative imaging protocols, optical imaging, employing constitutive luciferase reporters for bioluminescent imaging (BLI), has become the *de facto* technology for monitoring the growth and regression (in response to therapy). However, correlation of BLI intensity with tumor volume is poor (R^2^ = 0.6-0.8), particularly for larger tumors (>200 mm^3^) [44]. Problems arise due to inconsistent luciferin penetration of organs generally [45], and tumor specifically due to vascularity of the hypoxic tumor necrotic core [46], and this may be further exacerbated in a trial of pro-apoptotic therapies. Further, BLI intensity can be reduced by surrounding tissues, such as bladder with variable urine content [16] or incomplete fur removal [44], and thus volume estimates may be unreliable for prostate. Increasing tumor volume also directly reduces the ability of light to escape [14], and this imaging modality may be more properly described as qualitative [15].

While micro-computed tomography is lower cost than MR imaging, it cannot effectively distinguish prostate tumor from surrounding normal tissue [11]. In contrast, ultrasound imaging instrumentation is much more amenable to use in animal core facilities due to both acquisition and operating costs, as well as instrument size. Volume determinations using a micro-transrectal transceiver [41], has been shown to correlate well with volumes determined from the wet weight of dissected orthotopic prostate tumors, but are technically challenging. High frequency ultrasound measurements using the VisualSonics Vevo 770, that employs an external probe, show good correlation (r^2^ = 0.85) with dissected large autochthonous tumors [11]. Moreover, the relative ease of operation facilitates reasonable through-put, since scan times are on the order of 30 min including preparative anesthesia and depiliation. 2D-ultrasound allows in-plane sizing of tumors with a diameter as small as ~3 mm, which is within 10 % of the diameter of mouse prostate, as revealed by histology [35], but single slice imaging is subject to sampling error, few prostate tumor imaging studies report volume changes less than 10 mm^3^, and tumors with non-uniform shapes are poorly described by 2D-imaging. 3D-ultrasound for human prostate is a more precise and accurate methodology for determining prostate volume [47, 48]. High resolution 3D-ultrasound computed volumes from 0.5 to 10 mm^3^ correlate well with caliper measurements and histology for endometriotic cysts [45], and colorectal xenografts [34]. We therefore sought to exploit the precision of 3D-ultrasound by adapting it to accurately quantitate very small changes (as little as 3 % of a volume as small as ~10 mm^3^) in the mouse prostate under various physiological and pathological conditions. Figs. 1, 2 and 3 demonstrate that our use of the VisualSonics instrumentation and Amira 3D software for reconstruction allows very reproducible measurements of VP volume in intact mice (Fig. 1), mice undergoing castration induced regression (Fig. 2) and mice undergoing DHT supplementation induced re-growth (Fig. 3). In these imaging sessions the VP volumes varied from ~20 mm^3^ for intact animals to ~5 mm^3^ for regressed animals, emphasizing the high degree of accuracy in measuring very small glands. Because image processing involves operator-dependent segmentation of the raw US images, we performed blinded intra- and inter-operator assessments of reproducibility (Fig. 4) and found excellent agreement (CV ~3 %). We should note that assessment does not require extensive training in mouse anatomy, as individuals who had no prior experience were able to readily master the segmentation protocol within weeks. We also applied our methodology to two pathological animals models: prolactin transgene-driven benign prostatic hyperplasia (Fig. 5) and orthotopic implantation of human prostate cancer xenografts (Fig. 6). In both cases, we were able to detect pathological changes which increased the volumes by as little as 10 % (*c.f.* Fig. 6b, W3). In addition, the small size of the normal anterior prostate host site for implantation leads to variability when injecting tumor cells to establish prostate orthotopic xenografts, impairing reproducibility in the either the microenvironment or mis-location of the implantation and concomitant dissemination of the tumor in the peritoneum [49]. Thus, in contrast to other imaging modalities, which require large volume changes to be appreciated, this methodology reveals the magnitude of morphological changes more typically seen in human pathology, where the volumetric alterations are a small fraction of the original organ volume. Finally, in data not shown here, we have use the same protocol to image tumor formation in PTEN deficient mouse models of human prostate cancer and can similarly detect presumptive tumor which represents less than 10 % of prostate volume.

## Conclusions

We have developed an accurate, precise, and reproducible high frequency ultrasound imaging and 3D reconstruction protocol to serially quantitate prostate volume in live mice. This protocol allows determination of normal prostate growth and regression following hormone manipulation (ADT), as well as growth following androgen supplementation, in a model of BPH and in orthotopic prostatic tumor xenograft models. We anticipate that the utility of this technique can be extended to determining the efficacy of novel therapeutics in pre-clinical trials in mouse models of prostate cancer and benign prostatic hyperplasia.

## Abbreviations

PrCa: Prostate cancer
MRI-CHESS: Magnetic resonance imaging with chemical shift suppression
Pb-PRL: Probasin-driven prolactin
CV(s): Coefficient(s) of variation
RPMI: Roswell park memorial institute 1640 media
VP: Ventral prostate
DLP: Dorsal-lateral prostate
ADT: Androgen deprivation therapy
SEM: Standard error of the mean
BLI: Bioluminescent imaging
